# A novel AMPK activator, WS070117, improves lipid metabolism discords in hamsters and HepG2 cells

**DOI:** 10.1186/1476-511X-10-67

**Published:** 2011-04-29

**Authors:** Zeqin Lian, Yan Li, Jian Gao, Kai Qu, Jin Li, Linghua Hao, Song Wu, Haibo Zhu

**Affiliations:** 1 Institute of Materia Medica, Chinese Academy of Medical Sciences & Peking Union Medical College, Beijing, 100050, China

## Abstract

**Background:**

WS070117 is a novel small molecule compound that significantly improves lipid metabolism disorders in high-fat-diet (HFD) induced hyperlipidemia in hamsters.

**Methods and Results:**

We evaluated liver/body weight ratio, liver histology, serum and hepatic lipid content in HFD-fed hamsters treated with WS070117 for 8 weeks. Comparing with HFD fed hamsters, WS070117 (2 mg/kg per day and above) reduced serum triglyceride (TAG), total cholesterol (TC), low density lipoprotein cholesterol (LDL-C) and hepatic cholesterol and triglyceride contents. Oil Red O staining of liver tissue also showed that WS070117 improved lipid accumulation. We then carried out an experiment in the oleic acid (OLA)-induced steatosis model in HepG2 cell to investigate the lipid-lowering effect of WS070117. Oleic acid (0.25 mM) markedly induced lipid accumulation in HepG2 cells, but WS070117 (10 μM) inhibited cellular lipid accumulation. In OLA-treated HepG2 cells, WS070117 (above 1 μM) treatment reduced lipid contents which synthesized from [1-^14^C] labeled acetic acid. Because WS070117 is an analog of adenosine, we evaluated the effect of WS070117 on AMP-activated protein kinase (AMPK) signaling. The results showed that the activation of AMPK in OLA-induced steatosis in HepG2 cells was up-regulated by treatment with 0.1, 1 and 10 μM WS070117. The hepatic cellular AMPK phosphorylation is also up regulated by WS070117 (6 and 18 mg/kg) treatment in HFD fed hamsters.

**Conclusion:**

These new findings identify WS070117 as a novel molecule that regulates lipid metabolism in the hyperlipidemia hamster model. In vitro and in vivo studies suggested that WS070117 may regulate lipid metabolism through stimulating the activation of AMPK and its downstream pathways.

## Background

Metabolism syndromes are often linked to the macronutrient content of the diet and there is evidence that excessive consumption of macronutrients such as carbohydrates, fats, and even protein may eventually lead to the development of metabolic syndromes [1]. Diets high in fats and cholesterol have been demonstrated to induce weight gain, insulin resistance and hyperlipidemia in humans and animal models [2]. Control of serum lipids is important to prevent cardiovascular events. For many patients, however, achievement of tight serum lipid control by diet is difficult, requiring therapy with drugs [3]. Statins are one of the most widely used classes of drugs in clinical anti-hyperlipidemia therapy. However, availability of drugs that alleviate dyslipidemia syndromes is somewhat limited, warranting the discovery and characterization of novel molecules targeting various pathways involved in the pathogenesis of hyperlipidemia.

Activation of AMP-activated protein kinase (AMPK), a heterotrimeric energy-sensing protein, acts to restore cellular energy balance by promoting ATP-generating pathways such as fatty acid oxidation while simultaneously inhibiting ATP-utilizing pathways such as fatty acid synthesis [4]. Historically, the primary function of AMPK was to generate ATP when cellular levels are low. The AMPK system plays a major role in the regulation of hepatic glucose and lipid metabolism through its acute effects on energy metabolism and long-term effects on gene expression patterns in the liver [5]. Similarly, pharmacological activation of AMPK by metformin prevents intracellular accumulation of lipids, and reverses fatty liver in obese humans and mice [6,7]. Therefore, it is clearly an attractive therapeutic target in cardio-metabolic diseases.

During our screening for AMPK activators, we serendipitously identified WS070117 as a lead compound with potent lipid-regulating properties. The objective of this study was to evaluate the impact of WS070117 on an animal and cellular model of hyperlipidemia. Studies in the hamster hyperlipidemia model revealed that WS070117 markedly improved lipid abnormalities associated with conditions characteristic of the high fat diet (HFD)-induced lipid metabolism disorder. Investigations into cellular signaling mechanisms suggested that WS070117 may produce the lipid-regulating effects by the phosphorylation of the AMPK signaling pathway.

## Methods

### Synthesis of WS070117

**Figure 1:**
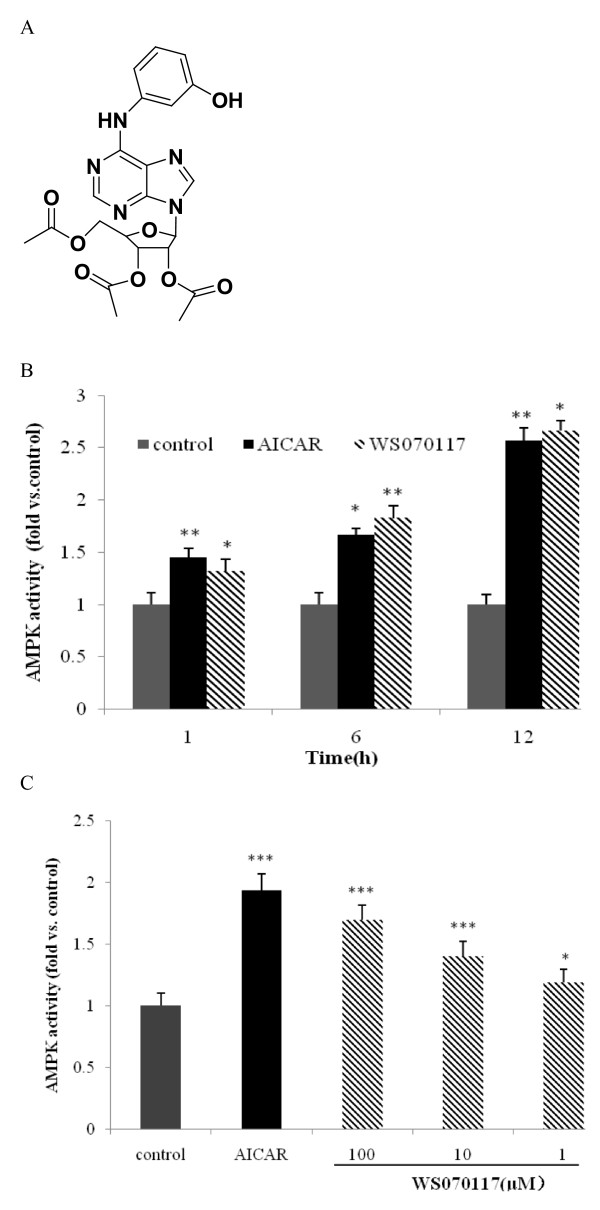
**A novel structure compound, WS070117, activated AMPK in HepG2 cells**. **A**: Structure of WS070117; **B**: Effect of WS070117 on AMPK activity in HepG2 cells detected by AMPK activity assays with SAMS peptide and [γ-32P]ATP used as substrates. HepG2 cells were treated with WS070117 (10 μM) for 1h, 6h and 12h. **C**: Effect of 12h treatment of WS070117 (1,10,100 μM) or AICAR (1 mM) on AMPK activity of HepG2 cells. AMPK activities are expressed relative to activity detected in HepG2 cells lysate. Each point is the mean (± SEM) of 3 separate experiments. **P < 0.01, *P < 0.05 as compared with control.

The synthesis of O^2'^,O^3'^,O^5'^-tri-acetyl-N_6_-(3-hydroxylaniline)adenosine, named as WS070117 (Figure 1A), began with acetylation of inosine using commercially available acetic anhydride, chlorination with SOCl_2_, and substitution with 3-aminophenol. Briefly, acetic anhydride was added to a suspension of inosine in dry pyridine at 0°C and stirred at room temperature for 6 h to yield the pure product O^2'^,O^3'^,O^5'^-tri-acetylinosine (Compound 1). Compound 1 was then dissolved in dry CH_2_Cl_2_:DMF (50:1) and a solution of SOCl_2 _in CH_2_Cl_2 _was added in a drop-wise manner. After dilution with CH_2_Cl_2_, the organic layer of the reaction mixture was washed with saturated NaHCO_3 _solution and brine and dried with anhydrous sodium sulfate. To a solution of O^2'^,O ^3'^,O^5'^-triacetyl-6-chloroadenosine in absolute ethanol was added 3-aminophenol and dry triethylamine. The mixture was then refluxed for 8 h at 60°C. The solution was concentrated in vacuum and the residue was chromatographed (ethyl acetate:petroleum ether, 2:1) to give WS070117 as a white solid.

### Animal

Golden hamsters (12-month-old) were purchased from Vital River Laboratory Animal Technology Co. Ltd. (Beijing, China). All animal treatment procedures described in this study were approved by the Animal Care and Use Committee at the Institute of Materia Medica, Chinese Academy of Medical Sciences and Peking Union Medical College (Beijing, China). Animals were housed under well-controlled conditions of temperature (22 ± 2°C), humidity (55 ± 5%) and a 12 h light-dark cycle with access to food and water *ad libitum*.

### Experiment design and drug administration

Except the control hamsters (n = 5), other hamsters (n = 40) were fed high fat diet (HFD) accustomedly for 2 weeks, blood was collected from orbit vein for assaying the content of serum total cholesterol and triglyceride for grouping. Then, The HFD fed hamsters were divided into the following groups with eight animals per group respectively, accounted into their body weight and the levels of serum total cholesterol and triglyceride, HFD model group, HFD+Simvastatin (2 mg/kg) as a positive group, HFD+WS070117 low dose group (2 mg/kg), HFD+WS070117 middle dose group (6 mg/kg) and HFD+WS070117 high dose group (18 mg/kg). The animals in normal control group were fed ordinary diet, and the other groups were still fed HFD *ad libitum *for 8 weeks with the indicated drugs oral administration. WS070117 was dissolved in distilled water prior to administration. All drugs were given once daily by oral gavage for 8 weeks. The animals in the normal control and in the HFD model control group received the same volum of distilled water as that of the drug-treated groups.

### Cell culture and treatment

The hepatic cell line HepG2 was purchased from the Cell Resource Center(IBMS, CAMS/PUMC, Beijing China) was grown in Dulbecco's modified Eagle's medium (DMEM, GIBCO, NY, USA) supplemented with 10% fetal bovine serum (FBS, MDgenics, St. Louis, MO, USA) and 1% penicillin-streptomycin under a humidified atmosphere of 5% CO_2 _in air. After growth to 70% to 80% confluence, cells were rendered quiescent by incubating with serum-free medium for 12 h before fatty acid treatment. For OLA-induced lipid deposition, the medium for cells at 75% confluence was exchanged for new medium with or without 0.25 mmol/L OLA (Sigma, St. Louis, MO, USA) [8]. Meanwhile, different concentration of WS070117 was added into medium and treated with OLA for 12h.

### Serum isolation and cholesterol determination

Blood samples were collected from the retro-orbital plexus 1 h after drug treatments. Serum was isolated at room temperature. Standard enzymatic methods were used to determine TC, TAG, LDL-c and high density lipoprotein cholesterol (HDL-C) levels with commercially available kits purchased from Bio Sino Bio-technology and Science Inc. (Beijing, China). Each sample was assayed in duplicate.

### Measurement of hepatic TAG and TC contents in hamster livers

100 mg of hamster liver tissue was taken for lipid extraction. Briefly, the frozen tissue was thawed and homogenized with a motor driven homogenizer in 2 ml chloroform/methanol (2:1). After homogenization, lipids were further extracted by rocking samples for 1 h at room temperature, followed by centrifugation at 5,000 rpm for 10 min. The liquid phase was washed with 0.2 volume of 0.9% saline. The mixture was centrifuged again at 2,000 rpm for 5 min to separate the two phases. The lower phase containing lipids was evaporated and lipids were dissolved in 0.5 ml isopropanol containing 10% Triton X-100 for TAG and TC measurements.

### Oil red O stain

For histological examination, pieces of liver tissues were fixed in 4% paraformaldehyde and dehydrated in 30% sucrose solution at room temperature. Tissues were then immersed in Optimal Cutting Temperature (OCT) solution on dry ice for Oil Red O staining. Three hamster livers were randomly picked from each group and three different tissue sections from each liver were processed and stained using routine laboratory procedures. For fat accumulation examination in HepG2 cells, culture dishes were washed with cold phosphate-buffered saline and fixed in 4% paraformaldehyde. After 2 changes of 60% isopropanol, Oil-Red-O was added with agitation for 10 minutes, followed by washing in 50% isopropanol. For each dish, 3 images were photographed, and a representative image is shown.

### AMP-activated protein kinase (AMPK) activity assay

AMPK activity assays were performed on lysates of HepG2 cells cultured in 6-well plates with 10% FBS/DMEM supplemented with 1,6,12 hours time course (10 μM) and 1,10,100 μM dosage (12 hours) of WS070117 by measurement of [γ-^32^P]ATP (Perkin-Elmer, Boston, MA, USA) radioactivity phosphorylated by a synthetic peptide substrate, SAMS [9]. AMPK activity was expressed as nmol of phosphate incorporated into the peptide substrate per min per mg of lysate (nmol/min/mg).

### Lipid synthesis assay

OLA HepG2 cells was co-incubated with 0.1,1 and 10 μM WS070117 for 12h. Incorporation of [1^-14^C] acetic acid (Perkin-Elmer, Boston, MA, USA) into lipid was performed as described previously [10]. After replacement with 1 ml of fresh medium, the cellular lipid was labeled by incubating for 2.5 h with [1^-14^C] acetic acid (0.2 μCi/ml). Lipid was extracted with n-hexane:2-propanol (3:2, v/v) and dried under a nitrogen stream. Aliquots of lipid samples were loaded onto thin-layer chromatography plates and chromatographed in a solvent mixture containing hexane-diethylether-acetic acid (85:30:1, v/v/v). Bands corresponding to triglyceride and cholesteryl ester were scraped off, immersed in non-aqueous scintillation fluid, and counted for radioactivity.

### Western blot analysis

The expression and phosphorylation of each protein were analyzed by Western blot analysis. Briefly, protein lysates were subjected to 10% SDS-polyacrylamide gel electrophoresis. Proteins were transferred to a nitrocellulose membrane and the membranes were blocked for 1 h at room temperature with 5% non-fat dry milk in Tris-buffered saline (TBS) containing 0.1% Tween 20. Immunostaining to detect each protein was achieved after over-night incubation (4°C) with a 1:1000 dilution of anti-phospho (thr-172) AMPK (1:2000), AMPK (1:2000) and anti-beta-actin (1:5000). Proteins were visualized after subsequent incubation with a 1:5000 dilution of anti-mouse or rabbit IgG conjugated to horseradish peroxidase and use of a Super Signal Chemical luminescence detection procedure. All antibodies used in this study were from Cell Signaling Technology Inc. (Beverly, MA, USA). Protein concentrations were determined using a bicinchoninic acid assay (Applygen Inc., Beijing China). Three independent experiments were performed for each condition and the intensity of immune-reactive bands was analyzed with a Bio-Rad calibrated densitometer.

### Statistical analyses

One-way ANOVA was used to determine significant differences among groups, after which the modified Student's t-test with the Bonferroni correction was used for comparison between individual groups. P < 0.05 was considered statistically significant.

## Results

### WS070117 increased AMPK activity

AMPK has been proposed to act as a fuel gauge in mammalian cells and to play a crucial role in regulating fat metabolism in the liver. The major pathways that regulate intracellular lipid metabolism are evidently present in HepG2 cells. The change of AMPK activity in HepG2 cells is strongly associated with intracellular lipid metabolism. Because WS070117 is an adenosine analogue, we studied the possibility that WS070117 could activate AMPK. Incubation of HepG2 cells with 1 ~ 100 μM WS070117 for 12h significantly activated AMPK, as measured in cell lysates. WS070117 also activated AMPK in a time dependently manner within 12 hours treatment. AICAR, one of the potent AMPK activators, also increased AMPK activity (Figure 1B, C).

### In vivo reductions of plasma and hepatic lipid contents by WS070117

**Figure 2:**
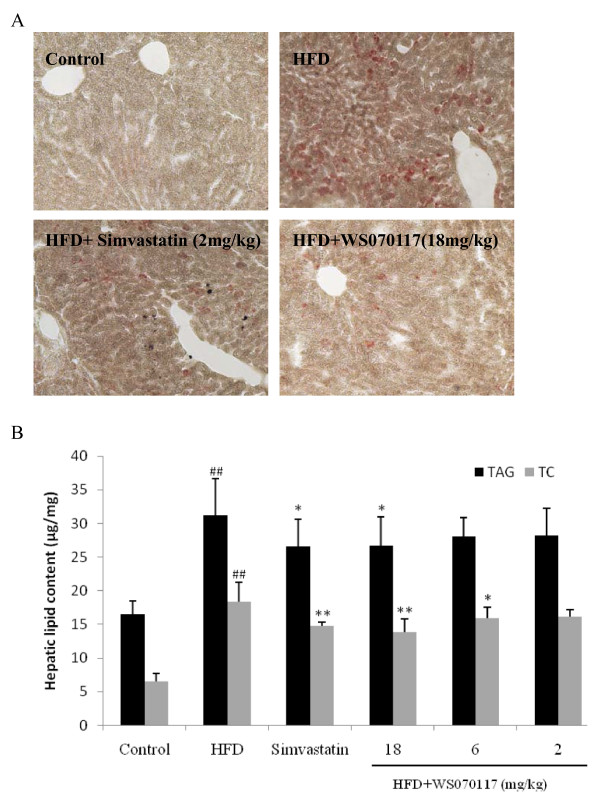
**Effects of WS070117 on hepatic lipids accumulation in HFD fed hamsters**. **A**: Representative frozen tissue sections of liver taken from hamsters fed chow diet, HDF diet, HFD+ simvastatin (2 mg/kg) and HFD+WS070117 (18 mg/kg) were stained with Oil Red-O to demonstrate the reduction in lipid droplets. **B**: Hepatic TC and TAG were measured in liver samples of control (n = 5), HFD, 8-weeks WS070117 (2, 6, 18 mg/kg) or simvastatin (positive control; 2 mg/kg) treated HFD fed hamsters (n = 8). **P < 0.01, *P < 0.05 as compared with HFD model. ^##^*P *< 0.01 as compared with control.

HFD with obese hamsters fed a diet containing 20% fat was used for producing the lipid metabolism dyslipidemia model. After 8 weeks, hamster body weights increased about 20% with no difference among the HFD treated groups. The liver/body weight ratio was markedly increased at the end of the experiment. The increases in the groups treated with WS070117 were significantly decreased the liver hypertrophy as compared with the HFD group (Table 1). WS070117 (2, 6 or 18 mg/kg/day) treatment resulted in significant decreases in serum TC, TAG and LDL-c as well as hepatic TAG and TC (Figure 2B).

**Table 1:** Hamsters were fed with normal chow or high fat diet (HFD) for 10 weeks

	Control(n = 5)	HFD(n = 8)		HDF+WS070117		HFD+Simvastatin 2 mg/kg(n = 8)
				
			2 mg/kg(n = 8)	6 mg/kg(n = 8)	18 mg/kg(n = 8)	
TAG (mmol/L)	2.05 ± 0.52	10.98 ± 5.84^##^	6.52 ± 2.75	5.91 ± 1.98*	4.51 ± 1.88**	6.18 ± 1.28*
TC (mmol/L)	3.95 ± 0.54	9.22 ± 3.87^##^	7.33 ± 1.84	6.81 ± 1.49	6.11 ± 0.65*	7.73 ± 1.31*
HDL (mmol/L)	1.48 ± 0.25	3.18 ± 1.46^#^	2.56 ± 0.89	2.45 ± 0.90	2.14 ± 0.26	2.51 ± 0.60
LDL (mmol/L)	2.40 ± 0.36	5.30 ± 2.01^##^	4.43 ± 0.58	3.97 ± 0.75*	3.16 ± 0.43**	2.51 ± 0.45*

Body weight	136 ± 16.5	168.8 ± 14.7^##^	166.5 ± 9.3	165.2 ± 15.7	164.5 ± 13.6	166.7 ± 12.6
Liver/Body weight	0.039 ± 0.0054	0.049 ± 0.0046^##^	0.044 ± 0.0056*	0.044 ± 0.0034*	0.045 ± 0.0023*	0.045 ± 0.0018*

Serum was separated after 8 weeks of WS070117 and Simvastatin treatment at the indicated doses from hamsters fed a high fat diet (HFD). The actual values of mean ± SD from 8 animals (control group, n = 5) are presented. ^##^*P *< 0.01, ^#^*P *< 0.05, as compared to vehicle control; ** *P *< 0.01, *P< 0.05 as compared to HFD model.

Oil Red O staining of livers showed that the hepatocytes of animals in the HFD group were compressed and separated by bulks of fat, demonstrating the abnormally high levels of fat accumulation in liver (Figure 2A). 8 weeks treatment with 18 mg/kg WS070117 substantially repressed these changes in HFD hamsters. Therefore, WS070117 helped to keep normal fat accumulation and deposition under HFD feeding conditions.

### WS070117 decreases lipid accumulation and de novo fatty acid synthesis in vitro

**Figure 3:**
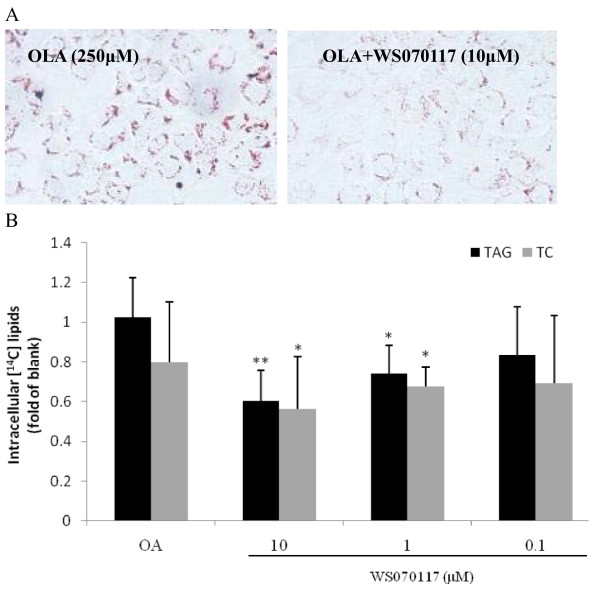
**Effects of WS070117 on steatosis and de novo lipids synthesis in OLA-HepG2 cells**. **A**: Oil Red O stain of 0.25 mM OLA-induced cellular lipid accumulation in WS070117 treated HepG2 cells. 12h treatment of WS070117 (10 μM) reduced the lipid droplets amount in HepG2 cells. **B**: TAG and TC synthesis was measured by the lipid accumulated from [1-^14^C] acetic acid, and was measured in control and WS070117 (0.1, 1, 10 μM) 12-h-treated OLA-HepG2 cells. **P < 0.01, *P < 0.05 as compared with OLA or HFD models.

Hepatic steatosis in humans is associated with accumulation of excess OLA and the end-product of de novo fatty acid synthesis [4]. AMPK-mediated phosphorylation of protein targets involved in cholesterol and fatty acids synthesis, however, lead to inhibition of cholesterol and TAG synthesis. In present study, Oil Red O staining showed that 10 μM WS070117 decreased the lipid accumulation in HepG2 cells induced by 250 μM OLA (Figure 3A). We then evaluated the impact of WS070117 on TAG and cholesterol synthesis by incubating HepG2 cells for 2 h with [^14^C]acetate in the presence of increasing concentrations of WS070117 (Figure 3B). Both cholesterol and TAG synthesis were dose-dependently inhibited by WS070117 (0.1 ~ 10 μM).

### Effect of WS070117 on phosphorylation and activation of AMPK in HepG2 cells and liver tissue

**Figure 4:**
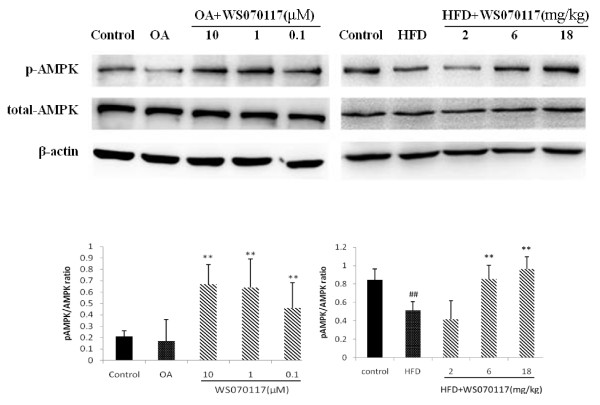
**WS070117 treatment increases AMP-activated protein kinase (AMPK) phosphorylation in OLA-induced HepG2 cells and HFD fed hamster livers. Data depict at least 3 experiments**. **P < 0.01 as compared with OLA and HFD groups. ^##^*P *< 0.01 as compared with control groups.

AMPK activation is thought to be a key proximal event in the cellular energy balance response, and AMPK phosphorylation levels are currently accepted as a marker of AMPK activity. Therefore, we determined the phosphorylation of AMPK in OLA overload HepG2 cells. After 12h treatment of WS070117, AMPK phosphorylation is activated at 0.1 10 and 1 μM. In HFD-fed hamster livers, AMPK phosphorylation was also increased by treatment with 6 or 18 mg/kg WS070117 (Figure 4).

## Discussion

The key finding of our present studies is that a novel synthetic small molecule, WS070117, alleviates dietary (i.e., HFD) induced lipid metabolism disorder in hamsters. Male Golden Syrian hamster, a widely used model in studying atherosclerosis and cholesterol metabolism in response to dietary fat manipulations, has many similar profiles in lipid metabolism compared to humans [11]. HFD that contains 20% saturated fat induces increases in hamster serum TAG, TC, LDL-C within 1 week; the higher levels reach a stable state after 4 weeks of treatment, as previously described [12,13]. After treatment for 8 weeks on a HFD (20% fat, 2% cholesterol), hamsters exhibit an obvious hyperlipidemia syndrome. WS070117 treatment markedly improved the impaired serum lipid content, suggesting that WS070117 regulates lipid metabolism to alleviate the hyperlipidemia. The chronic HFD causes caloric surplus. When the lipid supply exceeds energy consumption, a gradual accumulation of ectopic fatty acids begins. Excessive ectopic triglyceride deposition in liver can impair perfectly normal hepatocytes and then induce hepatic steatosis [14]. In the present study, the Oil Red O staining of liver sections showed significant lipid accumulation in HFD treatment groups. Long-term feeding (8 weeks) of the HFD induced severe hepatic steatosis. WS070117 treatment significantly suppressed the increase of liver fat accumulation that was evoked by the HFD. WS070117 treatment was also associated with a reduction in the gain in body mass in the HFD-fed hamsters. Serum glucose was increased after HFD treatment, but WS070117 showed no effect on glucose metabolism (data not shown).

The nucleus of the parent molecule from which WS070117 is derived is an analogue of adenosine. Previously, several groups demonstrated that adenosine is an effective activator of AMPK [15,16]. In the present in vitro studies, we found that WS070117 activated AMPK in HepG2 cells. AMPK is considered a master switch in regulating glucose and lipid metabolism. AMPK is an enzyme that works as a fuel gauge, being activated in conditions of high-energy phosphate depletion. In the liver, activation of AMPK results in decreased production of plasma cholesterol, triglyceride and enhanced fatty acid oxidation [17,18]. These facts suggested that WS070117 suppresses hepatic lipid accumulation possibly through activation of hepatocyte AMPK. HMG-CoA and acetyl CoA carboxylase (ACC), key enzymes in cholesterol and fatty acid synthesis, respectively, were the first enzymes shown to be phosphorylated and inactivated by AMPK [17,19]. In the cholesterol synthesis pathway, AMPK blocks the conversion of HMG-CoA to mevalonate. ACC is an important rate-controlling enzyme for the synthesis of malonyl-CoA, which is a critical precursor for biosynthesis of fatty acids. AMPK induced ACC phosphorylation is impaired in hepatocytes deleted of catalytic subunit, contributing to increased intracellular malonyl-CoA levels and triglyceride accumulation in the liver [20]. These findings demonstrated that WS070117 is an effective activator of AMPK with potential capability of inhibition of de novo hepatic lipogenesis.

To reinforce the lipid metabolism relevance of these cellular observations, we investigated the impact of WS070117 on OLA-treated HepG2 cells. Treatment of HepG2 cells with OLA induces morphological changes similar to those in steatotic hepatocytes [21,22]. Hepatic steatosis in humans is associated with accumulation of excess oleic acid, a monosaturated omega-9 fatty acid and the end-product of de novo fatty acid synthesis [23]. After WS070117 treatment, phospho-AMPK levels increased inside the cells, as demonstrated by Western blot experiments. As mentioned above, AMPK has been proposed to play a key role in the regulation of lipid metabolism. WS070117 significantly inhibited de novo hepatic cholesterol and triglyceride synthesis from [^14^C]acetate in OLA-treated HepG2 cells. When compared with the well-known AMPK activator AICAR, WS070117 induced a similar profile of effects on lipid synthesis, with inhibition of cholesterol and TAG synthesis. We conclude that activation of AMPK induced by WS070117 could be responsible for its lipid lowering profile.

Inhibition of hepatic lipogenesis is strongly associated with the anti-hepatic steatosis effect of WS070117. Endogenous OLA mediates the induction of de novo lipogenesis generally associated with intake of dietary saturated fat [24]. Reduced rates of de novo lipogenesis cause reduction of adipose stores. In the present study, we found that WS070117 treatment significantly decreased neutral lipid content accumulation in OLA-treated HepG2 cells. This result suggests that the inhibition of lipid synthesis on OLA-treated HepG2 cells by WS070117 treatment blocks the progress of hepatocyte steatosis. WS070117 also exhibited a similar stimulatory effect on AMPK phosphorylation of liver tissue in HFD-fed hamsters. Hence, the mechanism of action of WS070117 on diminution of hepatic fat storage in vivo appears to be similar to that which we demonstrated in the in vitro studies. We speculate that this decrease in hepatic lipid content mediated by AMPK could lead to the improved liver function in HFD-fed hamsters.

## Conclusion

The present studies suggest that WS070117 may be used as a newer pharmacological agent to treat or control HFD-induced lipid metabolism disorder. Our results also support the view that the anti-hyperlipidemia and anti-steatosis effects of WS070117 may be attributed to the activation of AMPK and inhibition of de novo hepatic lipogenesis.

## Competing interests

The authors declare that they have no competing interests.

## Authors' contributions

ZL and HZ conceived the idea and designed the study. LH and SW were responsible for synthesis of WS070117. ZL and JG performed animal experiments, did the sampling, prepared the samples and carried out Western bolting analyses. KQ and JL participated in the experiments related to AMPK enzyme activity assay. ZL carried out the lipid metabolism assay experiments in animals and cultured cell, performed the statistical analysis and interpretation of the data. ZL drafted the manuscript and YL provided critical corrections to the manuscript. All authors read and approved the final manuscript.
